# Patients’ understanding of telemedicine terms required for informed consent when translated into Kiswahili

**DOI:** 10.1186/s12889-018-5499-1

**Published:** 2018-05-03

**Authors:** Rachael Odhiambo, Maurice Mars

**Affiliations:** Department of TeleHealth, Nelson R Mandela School of Medicine, University of KwaZulu-Natal, Durban, South Africa

**Keywords:** Informed consent, Telemedicine, Language, Kiswahili, Bioethics

## Abstract

**Background:**

In Africa, where access to specialist medical services is often limited, telemedicine, the use of information and communication technologies for the provision of healthcare at a distance, can contribute towards enhancing access to healthcare. Informed consent is considered the cornerstone of ethical practice, especially when technology and techniques are considered new and or unproven. It is advised that informed consent should be gained in the patient’s mother tongue. However, many African languages have not kept pace with technology and lack the words and terms needed to describe computing and technical terms. Additionally, even when present, patients may not understand these words and terms. This affects the validity of informed consent given.

**Methods:**

Forty relevant computer terms and words used when explaining telemedicine during the consent process were selected and translated into Kiswahili. Patients at the outpatient department of the Kilifi County Hospital in Kenya participated. The study consisted of two phases. In the first, 50 people were asked whether they understood the translated words and terms and were able to explain their meaning. In the second (*n* = 42) they were asked to explain the meaning of the translated word, the original English word, and those words that could not be translated.

**Results:**

Of the 40 terms, 14 could not be translated (35%). A total of 92 people attending the Kilifi County Hospital participated. Their average age was 31.2 ± 10.6 y, 70.7% were female and 55.4% were from rural areas. More than half of the respondents did not understand videoconference, store and forward, digital photograph, wireless, World Wide Web, antivirus or email in either language. No-one understood the words telemedicine, firewall, encryption, decryption and tele-diagnosis.

**Conclusions:**

Currently it is unlikely that valid informed consent can be obtained for a telemedicine encounter in Kiswahili. Innovative solutions are required to overcome the barrier of languages failing to keep pace with technology and their effect on consent.

## Background

Telemedicine is defined by the World Health Organization (WHO) as the delivery of healthcare services, where distance is a critical factor, by all healthcare professionals using information and communication technologies (ICT) for the exchange of valid information [1]. Telemedicine uses two-way telecommunication technology, multimedia and computer networks to deliver or enhance healthcare [2]. Telemedicine is not new. Its history has followed the progress and evolution of ICT over time. As early as 1862 the telegraph was used to order medical supplies during the American Civil War [3] and for the management of casualties after an attack on the Barrow Creek telegraph station in Australia [4], followed by use of the telephone, radio, television, satellite links, computer networks, Internet, wireless networks and more recently the mobile phone [3].

Telemedicine has the potential of addressing the shortage of healthcare professionals in the developing World and improving access to quality medical care by allowing distant providers to evaluate, diagnose, treat and provide follow up care to patients in resource poor settings [5, 6]. Telemedicine is a feasible option to enhance access to healthcare services in Africa, improve quality of life and reduce the number of referrals from rural areas to specialists in urban areas thereby reducing travel costs to patients [1]. Despite the potential benefits of telemedicine, there are a number of challenges and barriers, one of which is the ethical issue of informed consent.

The minimum internationally agreed requirement for informed consent requires the treating healthcare professional to provide the patient or guardian, in the case of a child or patient who is not competent, with appropriate information about what is to occur so that they may make a voluntary and informed choice to accept or refuse treatment [1, 7]. The validity of consent is determined by it being done voluntarily, with adequate information having been provided, and the person consenting having a clear understanding of what it is to which they are consenting and the capacity to make the decision [8]. To reduce misunderstanding and ensure the validity of consent, information should be provided and consent gained in the patient’s or guardians’ native language. This is considered a critical requirement [9].

The issue of requiring informed consent for telemedicine has been debated. Some argue that it is no different to face to face consultation or existing methods of seeking a second opinion [10]. Others, feel that it is something new and unproven and patients should be protected by the use of written informed consent [11]. The American Telemedicine Association recommends informed consent as best practice and it is a requirement for many Telemedicine programs [10]. The World Medical Association’s “Statement on Guiding Principles for the Use of Tele-Health for the Provision of Health Care” states that relevant legislation and regulation that relate to patient decision making and consent, be it verbal, written, or recorded should be applied, and, “To the extent possible, informed consent shall be obtained by the physician before starting any service or intervention” and “Consent for telehealth should follow similar principles and processes as those used for other health services.” [12].

In South Africa, the Health Professionals Council, the statutory body tasked with regulating healthcare professionals requires that “Healthcare practitioners should not give medical advice or provide treatment using telemedicine without obtaining proper informed consent from the patient for both the treatment to be given and the use of telemedicine technology.” and that such consent should written, signed consent [13]. Obtaining “Proper consent” can be challenging for various reasons, one of which is language. Ideally consent should be obtained in the patient’s mother tongue.

There are over 7500 languages in the world with more than 2000 in Africa [14]. There is empirical evidence to indicate that language barriers directly affect healthcare delivery [15] and also interfere with the doctor-patient relationship, as patients who do not understand the doctor’s language are less likely to adhere to their prescribed medication and more likely to miss appointments than those who share a language with the doctors [16].

A recent study in South Africa reported the lack of information and communication technology words and terms in an indigenous language, isiZulu. These were words that would be required to explain telemedicine in order to obtain valid consent [17]. Only 7% of isiZulu first language speakers understood the sentence, “I consent to a telemedicine consultation” in their own language. In addition, when a technology literate health professional obtains consent for telemedicine from a technology naive patient through an interpreter, there is concern about the interpreter’s understanding of the words and terms, and their ability to explain these words and terms to the patient if there are no direct translations available in the indigenous language [18]. The ethical issues of not obtaining valid informed consent potentially impact on patient autonomy, the right to privacy and confidentiality, justice, fairness and the quality of care being compromised [19].

In Kenya healthcare services are provided by both public and private hospitals served by 10.7 skilled health professionals per 10,000 people [20] which is about one quarter of the health professionals considered necessary to achieve universal health coverage outlined in the 2030 Sustainable Development Goals [21]. The situation is further exacerbated by the unequal distribution of the health workers with 74% of the Kenyans living in rural areas [22] while a significant proportion of health professionals are based in the urban areas [23]. As a result, access to quality medical care and especially specialist care is limited in rural areas and referral often delays diagnosis and treatment [1, 24].

In recent years, there has been significant improvement in ICT infrastructure in Kenya [19]. Kenya has the highest Internet penetration in Africa, 89.4% compared to the African average of 31.2% [25]. In this environment, telemedicine presents a viable and cost effective approach to accessing quality healthcare in Kenya.

The aim of this study was to examine patients’ understanding of computing and technical terms required for explaining a telemedicine consultation in Kiswahili when obtaining informed consent. The objectives were to determine whether the technological words and terms could be translated into Kiswahili, whether these translations were understood in Kiswahili, whether untranslatable words were understood in English and the effect of this on gaining valid informed consent for a telemedicine encounter.

## Methods

Forty words and terms related to ICT and consent that could be required to explain synchronous (live videoconference based telemedicine) and asynchronous (store and forward telemedicine using email or a Web site) were selected from the list of terms used in a previous study [17]. These were translated from English to Kiswahili by a Kiswahili linguist, a Kiswahili teacher, a medical officer and an IT Specialist and back translated to check reliability. Words and terms that were not translatable were kept in the questionnaire. In addition to examining patients’ understanding of the technical words and terms and consent related words used, the questionnaire was also used to gather socio-demographic characteristics such as educational level, cell phone ownership or prior computer experience.

The study was undertaken at the Kilifi County Hospital (KCH) which is a referral hospital in Kilifi County in the Kenyan Coastal region. Most of the residents in this area belong to the Mijikenda community where Kiswahili is the most commonly spoken language. The overall literacy level is very low in Kenya 61.5% [26], and 65% in the coastal region [27]. A trained data collector visited the KCH out-patient department and used convenience sampling to invite Kiswahili speaking patients and accompanying helpers to participate. All participants gave written informed consent to participate.

Data were collected in two phases. In the first study participants were asked whether or not they understood the Kiswahili words and terms with a yes or no response. If they said they understood the word or term they were then asked to explain its meaning. In the second study, participants were again asked whether they understood and were able to explain the items/ terms in Kiswahili. They were then asked if they understood and were able to explain the meaning of the word in English. A sample size of 50 per study was considered adequate to determine understanding of the words.

Data were analysed using STATA13. Descriptive statistics were used to summarize the results, additionally univariate and bivariate analyses were conducted. The association between various variables was also conducted using chi-square tests with alpha set at 5%.

Permission to conduct the study was obtained from the Kilifi County Hospital ethics committee after obtaining an ethics clearance from Pwani University Institutional Review Board and the ethics committee of the University of Kwa-Zulu Natal.

## Results

A total 92 participants who attended the Kilifi County Hospital were interviewed. Their average age was 31.2 ± 10.6 y, 70.7% were female and 55.5% were from rural areas. Their level of education was low, 15.6% had never attended school, 50.0% had attended primary school, 30.4% secondary school and 4.3% had a college education. While 72.8% owned a mobile phone, 92.4% of the participants did not use computers.

Twenty-six (65%) of the words and terms could be translated. In some instances direct word to word translation to Kiswahili led to ambiguity with participants giving correct explanations of the word or term when used in another context. For example, ‘Autonomy’ was translated to ‘uhuru’ and most people said they understood the word ‘uhuru’ but what they actually meant from their explanation was ‘freedom’ which is true in Kiswahili but not in the required context. The word consent had more than one translation, ‘idhini’ - consent, permission, compliance, assent, approval, sanction and ‘ruhusa’ – permission [28].

In both the first and second phases of the study participants were asked if they understood the word or term in Kiswahili and were then asked to explain its meaning. The percentage of people who said that they understood the word, the percentage of those who having said they understood the word were able to explain the word and the overall percentage of participants who understood the word are shown in Table 1.

**Table 1:** Respondents understanding of words translated to Kiswahili (*n* = 92)

	Said understood	Said understood and correctly explained	% of sample who understood the translation
	n (%)	n (%)	%
antivirus	35 (38.0)	0 (0)	0
store and forward	32 (34.8)	0 (0)	0
realtime	20 (21.7)	0 (0)	0
intranet	14 (15.2)	0 (0)	0
non-realtime	5 (5.4)	0 (0)	0
world wide web	17 (18.5)	2 (11.8)	2.2
internet	26 (28.3)	3 (11.5)	3.3
wireless	29 (31.5)	11 (37.9)	12.0
digital signature	29 (31.5)	17 (58.6)	18.5
videoconference	41 (44.6)	18 (43.9)	19.6
digital photograph	37 (40.2)	24 (64.9)	26.1
email	41 (44.6)	31 (75.6)	33.7
trusted third party	78 (84.8)	35 (44.9)	38.0
authentication	68 (73.9)	36 (52.9)	39.1
computer	53 (57.6)	43 (81.1)	46.7
speakers	73 (79.3)	52 (71.2)	56.5
autonomy	89 (96.7)	62 (69.7)	67.4
network	78 (84.8)	62 (79.5)	67.4
microphone	78 (84.8)	69 (88.5)	75.0
confidentiality	82 (89.1)	71 (86.6)	77.2
consent	84 (91.3)	78 (92.9)	84.8
television	82 (89.1)	81 (98.8)	88.0
broadcasting	90 (97.8)	84 (93.3)	91.3
storage	89 (96.7)	84 (94.4)	91.3
security	92 (100)	89 (96.7)	96.7
video	90 (97.8)	89 (98.9)	96.7

In the second phase people were asked the meaning of the translated words and terms in Kiswahili and then their meaning in English. Their understanding of the words in Kiswahili, English, one or other language, both, or neither language are shown in Table 2.

**Table 2:** Respondents understanding of words and terms in Kiswahili and English (*n* = 42)

	Understood Kiswahili translation	Understood English word	Understood in neither language	Understood in both languages	Understood Kiswahili but not English	Understood English but not Kiswahili
	n (%)	n (%)	n (%)	n (%)	n (%)	n (%)
non-realtime	0	0	42 (100)	0	0	0
realtime	0	1 (2.4)	40 (95.2)	0	0	1 (2.4)
intranet	0	2 (4.8)	39 (92.9)	0	0	2 (4.8)
store and forward	0	2 (4.8)	39 (92.9)	0	0	2 (4.8)
antivirus	0	7 (16.7)	34 (81.0)	0	0	7 (16.7)
www	2 (4.8)	8 (19.0)	34 (81.0)	2 (4.8)	0	6 (14.3)
wireless	7 (16.7)	5 (11.9)	31 (73.8)	2 (4.8)	5 (11.9)	3 (7.2)
digital photograph	9 (21.4)	9 (21.4)	31 (73.8)	8 (19.0)	1 (2.4)	1 (2.4)
digital signature	9 (21.4)	9 (21.4)	30 (71.4)	7 (16.7)	2 (4.8)	2 (4.8)
videoconference	13 (31.0)	13 (31.0)	28 (66.7)	12 (28.6)	1 (2.4)	1 (2.4)
email	18 (42.9)	18 (42.9)	21 (50.0)	15 (35.7)	3 (7.1)	3 (7.1)
authentication	25 (59.6)	4 (9.5)	16 (38.1)	3 (7.1)	22 (52.4)	1 (2.4)
trusted third party	26 (61.9)	5 (11.9)	16 (38.1)	5 (11.9)	21 (50.0)	0
internet	0	29 (69.0)	13 (31.0)	0	0	29 (69.0)
confidentiality	28 (66.7)	13 (31.0)	12 (28.6)	11 (26.2)	17 (40.5)	2 (4.8)
microphone	35 (83.3)	28 (66.7)	7 (16.7)	28 (66.7)	7 (16.7)	0
consent	35 (83.3)	11 (26.2)	7 (16.7)	11 (26.2)	24 (57.1)	0
autonomy	36 (85.7)	3 (7.2)	6 (14.3)	3 (7.1)	33 (78.6)	0
speakers	32 (76.2)	37 (88.1)	5 (11.9)	32 (76.2)	0	5 (11.9)
computer	24 (57.1)	38 (90.5)	4 (9.5)	24 (57.1)	0	14 (33.3)
broadcasting	40 (95.2)	15 (35.7)	2 (4.8)	15 (35.7)	25 (59.6)	0
storage	37 (88.1)	31 (73.8)	2 (4.8)	28 (66.7)	9 (21.4)	2 (4.8)
television	36 (85.7)	40 (95.2)	1 (2.4)	35 (83.3)	1 (2.4)	5 (11.9)
video	41 (97.6)	40 (95.2)	1 (2.4)	40 (95.2)	1 (2.4)	0
security	42 (100)	34 (81.0	0	34 (81.0)	8 (19.0)	0
network	31 (73.8)	42 (100)	0	31 (73.8)	0	11 (26.2)

Half or more of the participants did not understand videoconference, store and forward, digital photograph, wireless, World Wide Web, antivirus or email in either language. The Kiswahili translation of Internet was not understood by anyone while 60% of people understood the English word, similarly, computer was better understood in English. The words, authentication, confidentiality, consent and autonomy were better understood in Kiswahili (59.6 – 85.7%) than English (7.2- 31%). Overall, more words were understood in Kiswahili than English. Respondents’ understanding of the 14 words that could not be translated is shown in Table 3.

**Table 3:** Understanding of the 14 English words that could not be translated (n = 42)

	Understood n (%)		Understood n (%)
firewall	0	synchronous	1 (2.4)
encryption	0	GPRS	4 (9.5)
decryption	0	hacker	4 (9.5)
latency	0	3G	5 (11.9)
telemedicine	0	electronic record	8 (19.0)
tele-diagnosis	0	wifi	11 (26.2)
electronic prescription	0	monitor	11 (26.2)

No-one understood the words telemedicine, firewall, encryption, decryption and tele-diagnosis

The translated words and terms were also classified into three groups, ICT, consent and entertainment terms (Table 4).

**Table 4:** Relationship of understanding of terms to: education level, gender and residence, computer usage and owning a cell phone

Group	Level of education	Residence	Gender	Computer use	Cell phone
ICT	< 0.001	< 0.001	0.003	0.019	0.032
Consent	< 0.001	< 0.001	0.009	< 0.001	< 0.001
Entertainment	< 0.001	0.291	0.037	0.001	0.125

There were significant associations between understanding of ICT and consent terms and level of education, gender (male), residence (urban), computer use, and mobile phone ownership. For the entertainment terms the relationships to residence and mobile phone ownership were not significant.

## Discussion

The key findings of this study were that few words and terms related to telemedicine and ICT technology were understood in either Kiswahili or English. Few people understood the words that could not be translated. Some translated words were understood, but in the wrong context, and while some claimed to understand the Kiswahili translations of technical terms and words their actual comprehension was generally poor: more than half of the translated words were understood by fewer than half of the respondents. Nobody understood the word telemedicine, which was not translatable. As such no-one would understand the common sentence in a consent form, “I consent to a telemedicine consultation.” To explain a telemedicine encounter requires use of words and terms such as Internet, email, videoconferencing, the World Wide Web, store and forward, and real-time, which were all poorly understood.

While it is considered advantageous to gain consent in the patient’s mother tongue this assumes that the words and terms used are understood. This was not so in either English or Kiswahili. Over half of the people in this study had only primary school education or no education. Although the medium for education is English fewer words were understood in English than Kiswahili. To date no study has been undertaken on doctors’ or interpreters’ understanding of translated words and their ability to explain these words.

The issue of consent for telemedicine remains unresolved. The World Medical Association’s stance is pragmatic but still assumes that consent gained is valid. Intuitively, it remains prudent to gain consent for a telemedicine encounter, but the validity of such consent must be questioned if people do not understand the words used to explain what is to happen, especially words and terms related to data transmission, security and storage. Regulators correctly see guidelines and regulations as ways of maintaining standard of care and protecting both patients and practitioners, but the reality is that in developing world countries overburdened doctors and nurses do not necessarily follow the rules and regulations regarding consent [29–31].

Regulators appear to be setting different standards for telemedicine, which is considered to be new, but it is not. The telephone has been used to seek and give advice on diagnosis and management since at least 1879 [32]. Doctors have not been required to ensure that the transmission of their voices over landlines are secure from interception so as to maintain confidentiality. Shared party lines were common, but this was not seen as a major impediment to the use of ICT for the provision of health services. Likewise, doctors have written letters seeking advice and sent reports about patients by paper based mail for centuries. They were not, and are still not required to ensure that their mail could not be read or intercepted by anyone else, but end to end encryption is considered mandatory for email communication of patient information.

This is the second study to investigate patients’ understanding of words and terms used to explain telemedicine when gaining consent in an indigenous language. The findings of this study are similar to the South African study, where only 7% of isiZulu speakers understood the isiZulu words for consent and telemedicine in the sentence, “I consent to a telemedicine consultation” [17]. Many more people understood the Kiswahili word for consent but no-one understood telemedicine. These two studies raise more questions than answers. Telemedicine facilitates access to care but how is valid informed consent for its use to be obtained?

The issue of understanding telemedicine and its associated terms and the effect of this on the validity of consent raises an ethical dilemma. If telemedicine is available and provides rapid access to a specialist and or a level of care not available locally is it ethical for a doctor not to use it because consent may not be truly informed? Failure to use telemedicine because of inability to obtain valid consent might impose a lower quality of care on the patient.

There is need to find alternative ways explain the concepts of telemedicine and associated technology issues in a way and in language that people understand. We need to go back to the drawing board – the use of comic book cartoon sequences of what occurs in, and needs to be understood about, a telemedicine encounter may be a novel way forward. Pragmatic solutions are required.

## Conclusion

Telemedicine improves access to medical care for rural, remote and underserved communities. This is especially important in the developing world. Currently it is unlikely that valid informed consent can be obtained for a telemedicine encounter in Kiswahili. Innovative solutions are required to overcome the problem, of languages failing to keep pace with technology and the effect of this on consent. Problems of consent should not block its use. Simplification of the consent process would be of great benefit for both the patient and health service provider [33].

## Abbreviations

ICT: Information and communication technologies
KCH: Kilifi county hospital
WHO: World Health Organization
